# Vitamin D Adequacy Conditions the Prolactin-Suppressive Effect of Metformin in Men Receiving Prolactin-Elevating Medications

**DOI:** 10.3390/nu18071062

**Published:** 2026-03-26

**Authors:** Robert Krysiak, Karolina Kowalcze, Giovanni Cangelosi, Andrea Deledda, Bogusław Okopień

**Affiliations:** 1 Department of Internal Medicine and Clinical Pharmacology, Medical University of Silesia, Medyków 18, 40-752 Katowice, Poland; bokopien@sum.edu.pl; 2 Department of Pediatrics in Bytom, Faculty of Health Sciences in Katowice, Medical University of Silesia, Stefana Batorego 15, 41-902 Bytom, Poland; kkowalcze@sum.edu.pl; 3 Department of Pathophysiology, Faculty of Medicine, Academy of Silesia, Rolna 43, 40-555 Katowice, Poland; 4 School of Pharmacy, Experimental Medicine and “Stefania Scuri” Public Health Department, University of Camerino, 62032 Camerino, Italy; giovanni01.cangelosi@unicam.it; 5 Endocrinology and Obesity Unit, Department of Medical Sciences and Public Health, University of Cagliari, 09124 Cagliari, Italy; andredele@tiscali.it

**Keywords:** antipsychotics, men, metformin, pituitary, prolactin excess, vitamin D

## Abstract

**Background/Objectives**: Metformin has been proposed as a potential treatment for hyperprolactinemia irrespective of etiology. Previous studies suggest that vitamin D deficiency attenuates the prolactin-lowering effect of metformin in women. This study examined whether vitamin D status modifies the effects of this agent on prolactin and other anterior pituitary hormones in men with iatrogenic hyperprolactinemia. **Methods**: Seventy-five adult men with antipsychotic-induced hyperprolactinemia and type 2 diabetes or prediabetes were enrolled. Participants were assigned to three equal groups based on vitamin D status and supplementation: vitamin D-naive men with sufficient levels (group 1), vitamin D-naive men with deficiency (group 2), and men with sufficient vitamin D levels receiving oral supplementation for at least six months (group 3). All participants received metformin (3 g/day) for six months. Plasma 25-hydroxyvitamin D, markers of glucose metabolism, total and monomeric prolactin, TSH, gonadotropins, ACTH, testosterone, and IGF-1 were measured at baseline and after treatment. **Results**: Baseline characteristics were comparable among groups except for 25-hydroxyvitamin D levels. Seventy participants completed the study. Metformin improved glycemic control and insulin sensitivity in all groups, with greater effects in men with sufficient vitamin D status. Reductions in total and monomeric prolactin were observed only in groups 1 and 3 and were associated with baseline prolactin concentrations and pretreatment 25-hydroxyvitamin D levels. These changes were accompanied by modest increases in LH and testosterone, and improvements in sexual functioning. Vitamin D levels and other hormonal parameters remained unchanged. The magnitude of the metformin effect did not differ between groups 1 and 3. **Conclusions**: Adequate vitamin D status is necessary for metformin to reduce prolactin levels in men with iatrogenic hyperprolactinemia.

## 1. Introduction

The anterior pituitary gland is a target tissue for metformin. Administration of this agent has been shown to suppress the secretion of several adenohypophyseal hormones, including thyroid-stimulating hormone (TSH) [1,2], follicle-stimulating hormone (FSH) [3], luteinizing hormone (LH) [4], and prolactin (PRL) [5,6,7,8,9,10,11,12,13]. Of particular interest, the PRL-lowering effect of metformin has been consistently demonstrated across diverse etiologies of hyperprolactinemia, including PRL-secreting pituitary adenomas [7,9], iatrogenic hyperprolactinemia [7,8,10,12,13], empty sella syndrome [7], traumatic brain injury [7], polycystic ovary syndrome-associated PRL excess [5,6], and idiopathic hyperprolactinemia [7]. The effect of metformin on lactotroph secretory function appears to be multifactorial and modulated by several biological and clinical determinants. First, the magnitude of PRL reduction depends on baseline hormone concentrations, with the most pronounced effects observed in individuals with markedly elevated pretreatment PRL levels; in contrast, no significant effect has been demonstrated in normoprolactinemic subjects [7]. Second, metformin exerts isoform-specific effects on PRL, selectively reducing the biologically active monomeric fraction while sparing high-molecular-weight polymeric PRL (macroprolactin) [14]. Third, sex-related differences have been reported, with a more pronounced inhibitory effect on PRL secretion in women than in men [15]. Lastly, the effect appears to be dose-dependent, with greater reductions observed in patients receiving higher doses of metformin [8]. In addition to reducing elevated PRL levels as monotherapy, the drug enhanced the PRL-lowering effects of dopamine agonists, which represent the treatment of choice for the pharmacological management of hyperprolactinemia [6].

Among the multiple etiologies of hyperprolactinemia, metformin has emerged as a potentially valuable therapeutic option for the management of antipsychotic-induced PRL excess. Several strategies have been proposed to reduce PRL concentrations in affected patients, including dose reduction or discontinuation of the causative antipsychotic, switching to PRL-sparing agents, adjunctive aripiprazole therapy, and the use of dopamine agonists [16,17]. Nevertheless, most of these approaches—apart from adjunctive aripiprazole—are associated with a substantial risk of exacerbating psychotic symptoms or triggering disease relapse [16,17]. Adjunctive aripiprazole, in turn, carries a risk of potentiating other antipsychotic-induced adverse effects [10]. Additional considerations further support the use of metformin in this patient population. Antipsychotics are predominantly prescribed for the treatment of schizophrenia and bipolar disorder, conditions that are inherently associated with an increased risk of type 2 diabetes, prediabetes, and metabolic syndrome [18,19,20]. This metabolic vulnerability is further compounded by antipsychotic treatment itself [21,22]. As a result, a considerable proportion of patients receiving long-term antipsychotic therapy already meet metabolic indications for metformin, reinforcing its potential dual benefit in this population.

The incidence of schizophrenia is higher among men than women, with a ratio of nearly 1.4:1 [23]. In addition, schizophrenia has an earlier onset in males—by approximately 3–4 years—than in females, and men have been found to be at greater risk of experiencing prominent negative symptoms [23]. Accumulating evidence indicates that the response to antipsychotic treatment differs by sex, with women generally demonstrating greater therapeutic responsiveness but also a higher susceptibility to treatment-related adverse effects [24]. Thus, males and females may differ in treatment details and in PRL responses to these agents. The majority of studies reporting a PRL-lowering effect of metformin in iatrogenic hyperprolactinemia have included mixed-sex cohorts [10,12] or exclusively female populations [7,8]. To date, only two studies—both conducted by our research group—have specifically examined the effects of metformin on lactotroph secretory function in men, and their findings were inconclusive [14,15]. While these inconsistencies may be partly attributable to differences in the etiology of PRL excess or baseline testosterone concentrations, the influence of additional modifying factors cannot be excluded, thereby highlighting the need for further investigation. Vitamin D status may represent one such modifier of metformin action in men. Patients with schizophrenia have been found to exhibit lower serum concentrations of 25-hydroxyvitamin D (25OHD) compared with healthy controls and to be at increased risk of vitamin D deficiency or insufficiency [25]. Moreover, improvements in extrapyramidal symptoms among patients with schizophrenia have been shown to correlate directly with serum 25OHD levels [26], and vitamin D supplementation has been associated with favorable effects on lipid profiles in individuals receiving olanzapine [27]. Notably, vitamin D status has also been reported to modulate the PRL-lowering effect of metformin in women [28]. Thus, the primary aim of this study was to compare the effects of metformin on lactotroph secretory function in men with antipsychotic-induced hyperprolactinemia stratified by low versus normal vitamin D status.

## 2. Materials and Methods

All study procedures were conducted in accordance with internationally recognized ethical guidelines for research involving human participants. Prior to enrollment, participants provided written informed consent after receiving comprehensive information about the study objectives, potential risks, and anticipated benefits. Ethical approval for the study protocol was obtained from the appropriate institutional review committee.

### 2.1. Study Population

**Figure 1:**
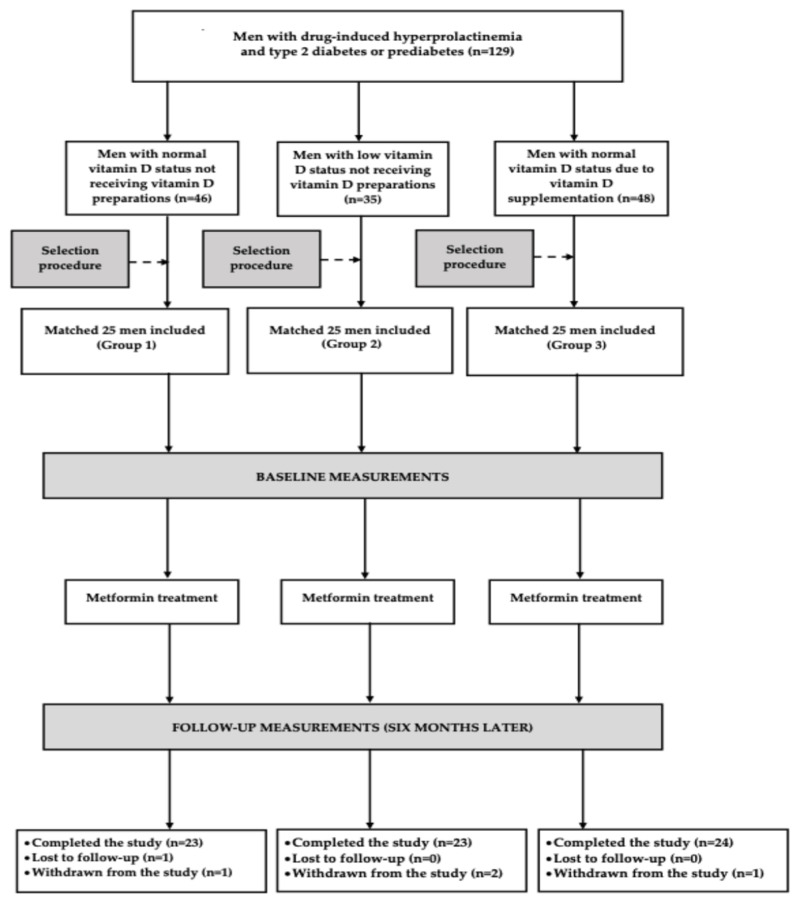
Flow the patients through the study.

The study employed a prospective, matched cohort design. Enrollment was limited to men aged 25–70 years with hyperprolactinemia secondary to antipsychotic treatment. Patients were required to be treated with either a first-generation or a second-generation antipsychotic carrying a high risk of inducing hyperprolactinemia. Individuals receiving atypical antipsychotics with a low likelihood of increasing prolactin concentrations were excluded to minimize the possibility that hyperprolactinemia could be attributed to factors other than antipsychotic therapy. The severity of schizophrenia-related symptoms was assessed using the six-item version of the Positive and Negative Syndrome Scale (PANSS-6). Eligibility required continuous treatment with unchanged antipsychotic dosages for a minimum of 12 weeks, as well as documentation of total PRL concentrations exceeding 40 ng/mL on two separate assessments conducted at least four weeks apart. Eligibility further required the presence of type 2 diabetes or prediabetes inadequately controlled by lifestyle intervention alone for at least 12 weeks, thereby qualifying participants for initiation of metformin therapy. The diagnosis of diabetes and prediabetes was made in accordance with standard clinical criteria [29]. The study participants (*n* = 75) were assigned to one of three groups, each comprising 25 patients. Group 1 included vitamin D-naive men with normal vitamin D status, defined as 25OHD concentrations between 75 and 150 nmol/L. Group 2 included men with low vitamin D status, defined as 25OHD concentrations between 25 and 75 nmol/L. Patients were included in this group only if they declined vitamin D supplementation. Group 3 comprised men with normal vitamin D status who had been receiving oral vitamin D supplementation (25–100 μg daily) for at least six months due to prior vitamin D deficiency or insufficiency. Low vitamin D status encompassed both vitamin D deficiency (25OHD < 50 nmol/L) and vitamin D insufficiency (25OHD 50–75 nmol/L), as proposed by the Endocrine Society and the World Medical Association [30,31]. Participants with 25OHD levels below 25 nmol/L were excluded for ethical reasons, as this threshold is widely recognized as indicative of severe vitamin D deficiency, which is associated with an increased risk of osteomalacia, falls, fractures, and myopathy [32,33]. The lower limit of normal 25OHD (75 nmol/L) corresponded to the threshold concentration to be achieved during supplementation with exogenous vitamin D, while the upper limit of normal (150 nmol/L) was established as the upper boundary of the target range, above which the risk of potential adverse effects increases [30]. Before study initiation, statistical modeling was used to determine the minimum sample size required to detect a clinically meaningful 20% change in total PRL, the primary outcome measure. Assuming a statistical power of 80% and a two-sided type I error rate of 5%, the analysis indicated that 20 participants per group were required. The effect size and pooled standard deviation (0.5) were chosen based on the results of our previous study assessing the impact of metformin on prolactin concentrations in men with hyperprolactinemia [34]. Additionally, they were chosen to minimize the risk of false-positive results arising from physiological fluctuations in hormone levels, as well as variations associated with differing levels of physical and emotional stress, sleep adequacy, and sexual activity [35,36]. To account for potential attrition, the planned sample size was increased by 25% for each group. All study groups were selected from larger pools of eligible male candidates using a computerized matching procedure to ensure balance with respect to age, insulin sensitivity, and baseline PRL concentrations (Figure 1). Seasonal variation in circulating 25-OHD levels was addressed by distributing recruitment evenly throughout the year, ensuring comparable numbers of participants were enrolled during each season.

Individuals were excluded from the study if they had poor glycemic control (HbA_1c_ > 8.0%), hyperprolactinemia of mixed or alternative etiology, or macroprolactinemia. Additional exclusion criteria included the presence of endocrine or autoimmune disorders, malabsorption syndromes, renal or hepatic insufficiency, other serious disorders (except for schizophrenia and bipolar disorder), or the use of medications known to interact with metformin or antipsychotic agents or to interfere with endocrine laboratory assessments.

### 2.2. Study Design

Individual dietary questionnaires were also analyzed to assess calorie and macronutrient intakes. During the study, only the principal investigator was aware of patients’ baseline vitamin D concentration, which determined cohort assignment. Such information was not available to the staff seeing the patients during visits, and the dietary diaries did not record the use or dosage of vitamin D supplements. This approach was implemented to minimize potential observer bias [37]. Throughout the study, all participants received metformin therapy and were instructed to adhere to the prescribed dietary recommendations. During the first week, metformin was administered at a dose of 850 mg twice daily; over the subsequent two weeks, the dose was uptitrated to 1000 mg twice daily. Beginning in week 4, the total daily dose was increased to 3000 mg, administered in three divided doses. To reduce the likelihood of gastrointestinal adverse effects, metformin was taken with or immediately after meals. To minimize the potential for pharmacokinetic interactions, metformin and exogenous vitamin D supplements, if taken, were administered at least four hours apart. No modifications to antipsychotic therapy were permitted throughout the entire study period. Furthermore, all patients in group 3 continued to receive exogenous vitamin D supplementation at the same dosage as before the study. The short-term use of antimicrobials, nonsteroidal anti-inflammatory drugs, acetaminophen, antitussives, antidiarrheal agents, laxatives, or soporifics was permitted, provided that treatment did not exceed one week and was discontinued at least four weeks before the final study visit. Treatment adherence was evaluated at each follow-up visit, conducted every eight weeks, through participant interviews and pill counts. Compliance with nonpharmacological recommendations was assessed by analysis of individual dietary questionnaires. Total daily vitamin D intake was estimated from individual diaries using food composition tables, with vitamin D intake from all dietary sources summed accordingly. Moreover, at both the beginning and the end of the study, all enrolled patients completed a questionnaire assessing sexual function. Assessment was limited to the erectile function and sexual desire domains of the International Index of Erectile Function-15 (IIEF-15) questionnaire for heterosexual men [38]. The remaining domains were not completed, as sexual function assessment served as a secondary endpoint of the study.

### 2.3. Laboratory Assays

Fasting venous blood samples were obtained from the antecubital vein between 07:30 and 08:30 h after a minimum fasting period of 12 h. Participants remained seated and at rest for at least 30 min prior to venipuncture. In view of the pulsatile nature of PRL secretion and its susceptibility to stress-related fluctuations [39], PRL concentrations were determined using three sequential blood samples collected at 20 min intervals. All biochemical measurements were performed in duplicate during a single analytical run, and the mean of the two measurements was used for statistical analysis. Laboratory personnel were unaware of both the participants’ identities and the order of study procedures. Plasma glucose levels and whole-blood HbA_1c_ were analyzed using a COBAS Integra analyzer (Roche Diagnostics, Basel, Switzerland). Plasma concentrations of insulin, PRL, TSH, gonadotropins, and testosterone were measured by chemiluminescent immunoassays based on acridinium ester technology (ADVIA Centaur XP, Siemens Healthcare Diagnostics, Munich, Germany). PRL measurements were performed both before and after polyethylene glycol precipitation to distinguish total and monomeric fractions. Concentrations of adrenocorticotropic hormone (ACTH) and insulin-like growth factor 1 (IGF-1) were quantified using solid-phase chemiluminescent immunometric assays (Immulite, Siemens, Munich, Germany). Insulin resistance was estimated using the homeostasis model assessment (HOMA-IR), calculated according to the formula: fasting insulin (mU/L) × fasting glucose (mg/dL)/405.

### 2.4. Statistical Analysis

All variables were log-transformed to achieve approximately Gaussian distributions. Comparisons between groups at the same time point were performed using one-way analysis of variance, followed by Bonferroni’s post hoc test. Baseline measurements were compared with follow-up measurements within each treatment group using paired *t* tests. Dichotomous or nominal variables were compared using χ^2^ tests. Relationships between the assessed variables were examined by computing bivariate correlations using Pearson’s *r*. In all analyses, differences were considered statistically significant when two-tailed *p* values were <0.05.

## 3. Results

During the study, two individuals (one assigned to group 1 and one to group 2) were withdrawn due to metformin-related gastrointestinal adverse events (diarrhea, nausea, vomiting, abdominal pain, and bloating). These symptoms resolved completely after discontinuation of the treatment. In addition, two male participants from groups 2 and 3 discontinued participation following changes in their antipsychotic regimens. One male participant in group 1 was lost to follow-up for reasons unrelated to the study protocol. Data from the 70 participants who completed the study were included in the final analysis, as compliance with the intervention was considered acceptable. A post hoc power assessment indicated that the sample size was sufficient.

**Figure 2:**
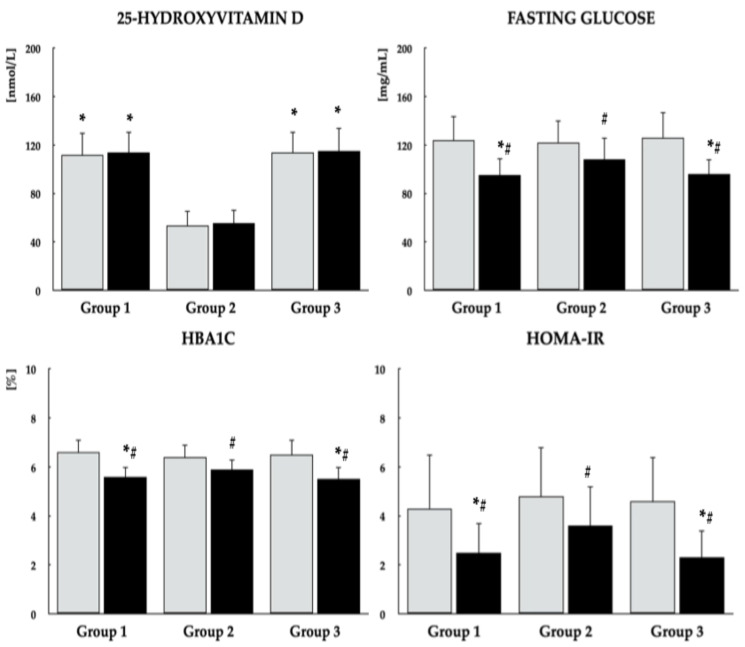
Effect of metformin on 25-hydroxyvitamin D levels and markers of glucose homeostasis in men with antipsychotic-induced hyperprolactinemia across different vitamin D statuses. Data are presented as mean ± standard deviation. Gray bars represent values at baseline; black bars represent values at study completion. Group 1: men with normal vitamin D status not receiving vitamin D preparations; Group 2: men with low vitamin D status not receiving vitamin D preparations; Group 3: men with normal vitamin D status due to vitamin D supplementation. * *p* < 0.05 vs. the corresponding value in group 2. ^#^ *p* < 0.05 vs. baseline values within the same group.

**Figure 3:**
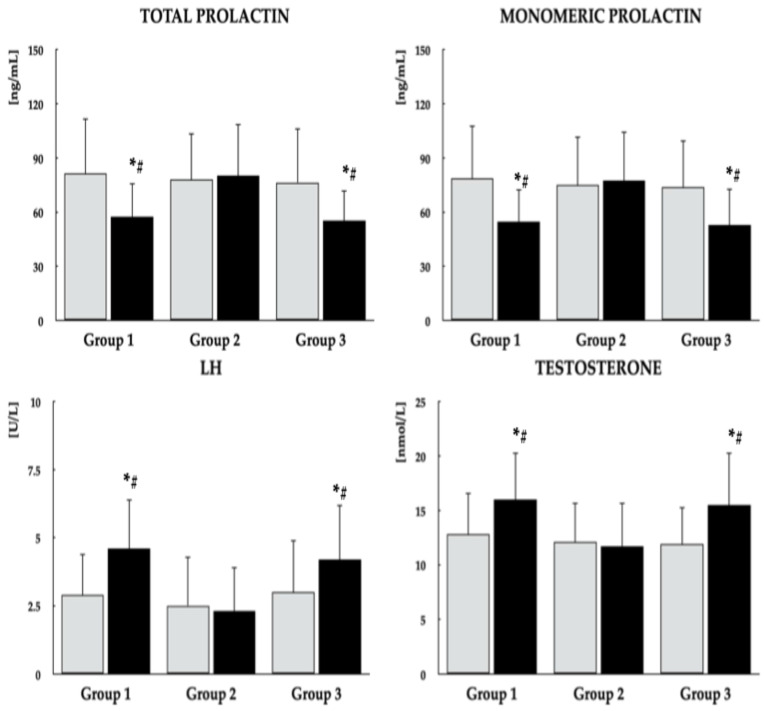
Effect of metformin on plasma prolactin, LH and testosterone in men with antipsychotic-induced hyperprolactinemia across different vitamin D statuses. Data are presented as mean ± standard deviation. Gray bars represent values at baseline; black bars represent values at study completion. Group 1: men with normal vitamin D status not receiving vitamin D preparations; Group 2: men with low vitamin D status not receiving vitamin D preparations; Group 3: men with normal vitamin D status due to vitamin D supplementation. * *p* < 0.05 vs. the corresponding value in group 2. ^#^ *p* < 0.05 vs. baseline values within the same group.

**Figure 4:**
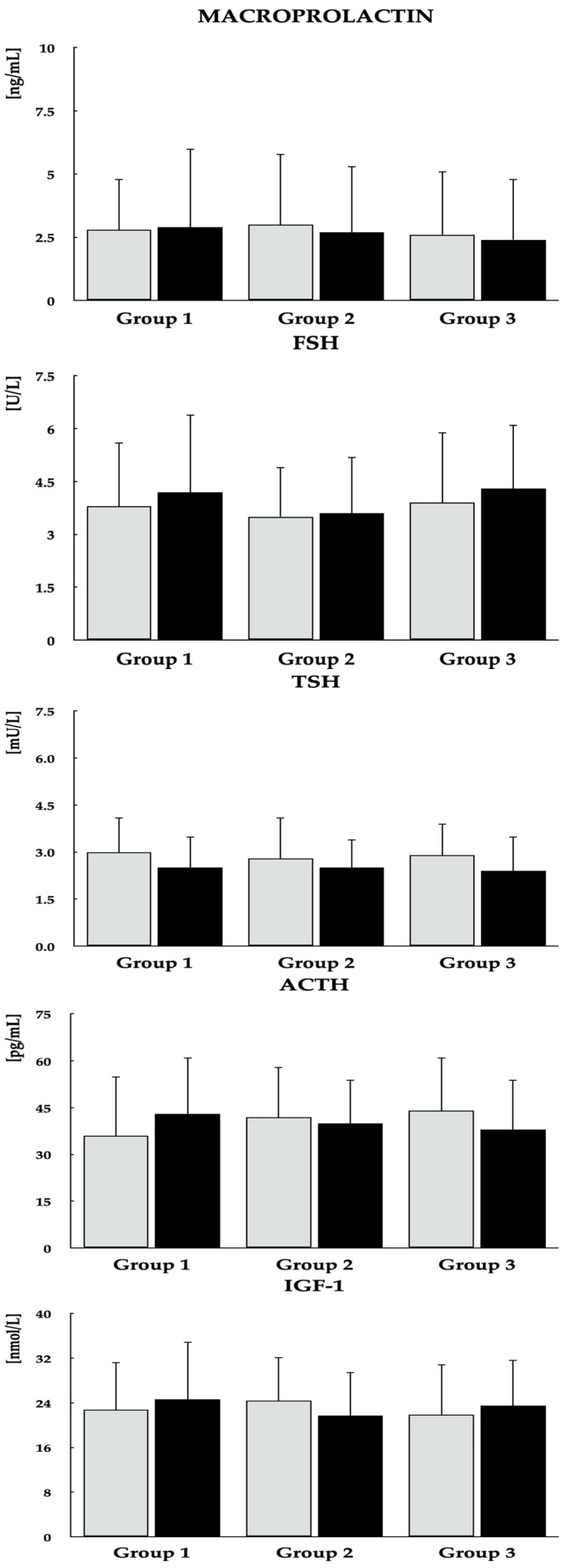
Effect of metformin on the remaining assessed parameters in men with antipsychotic-induced hyperprolactinemia across different vitamin D statuses. Data are presented as mean ± standard deviation. Gray bars represent values at baseline; black bars represent values at study completion. Group 1: men with normal vitamin D status not receiving vitamin D preparations; Group 2: men with low vitamin D status not receiving vitamin D preparations; Group 3: men with normal vitamin D status due to vitamin D supplementation.

Baseline comparisons revealed no significant differences among the study groups with regard to age, smoking status, educational attainment, occupational status, type of work performed, physical activity, the proportion of patients with type 2 diabetes/prediabetes, PANSS-6 score, antipsychotic treatment, body mass index, or blood pressure. Dietary vitamin D intake from food without vitamin D in tablet/capsule form was higher in group 1 compared with the other two groups (Table 1). The mean daily dose of vitamin D in the tablets or capsules used by patients in group 3 was 45.7 ± 19.0 µg. The mean total vitamin D intake (from both dietary sources and tablets/capsules) in this group was 55.2 ± 18.3 µg and was higher than the dietary vitamin D intake in groups 1 and 2. Mean calorie and macronutrient intakes did not differ between the study groups (Supplementary Table S1). Except for 25OHD, there were no differences in the measured parameters (Figure 2, Figure 3 and Figure 4). As shown in Table 2, both the mean daily doses and the individual antipsychotic dosages were similar across all study groups. On the final study day, PANSS-6 scores were 9.6 ± 2.1, 10.2 ± 2.3, and 9.9 ± 2.0 in groups 1, 2, and 3, respectively, with no significant between-group differences or within-group changes from baseline.

The total daily dietary intake of vitamin D during the study in group 1 was 19.2 ± 7.0 µg, which was higher than in group 2 (9.4 ± 4.6 µg) and group 3 (9.7 ± 3.5 µg); however, in none of the groups did it differ from the intake observed before the initiation of metformin treatment. Because patients were required to maintain an unchanged intake of vitamin D from tablets and capsules, the total vitamin D intake during the study in group 3 was 55.4 ± 18.8 µg. This value did not differ from the baseline intake in this group and was higher than the intake observed in the other two groups, which did not receive supplementation. Calorie and macronutrient intakes across all study groups remained stable throughout the study period (Supplementary Table S1).

**Figure 5:**
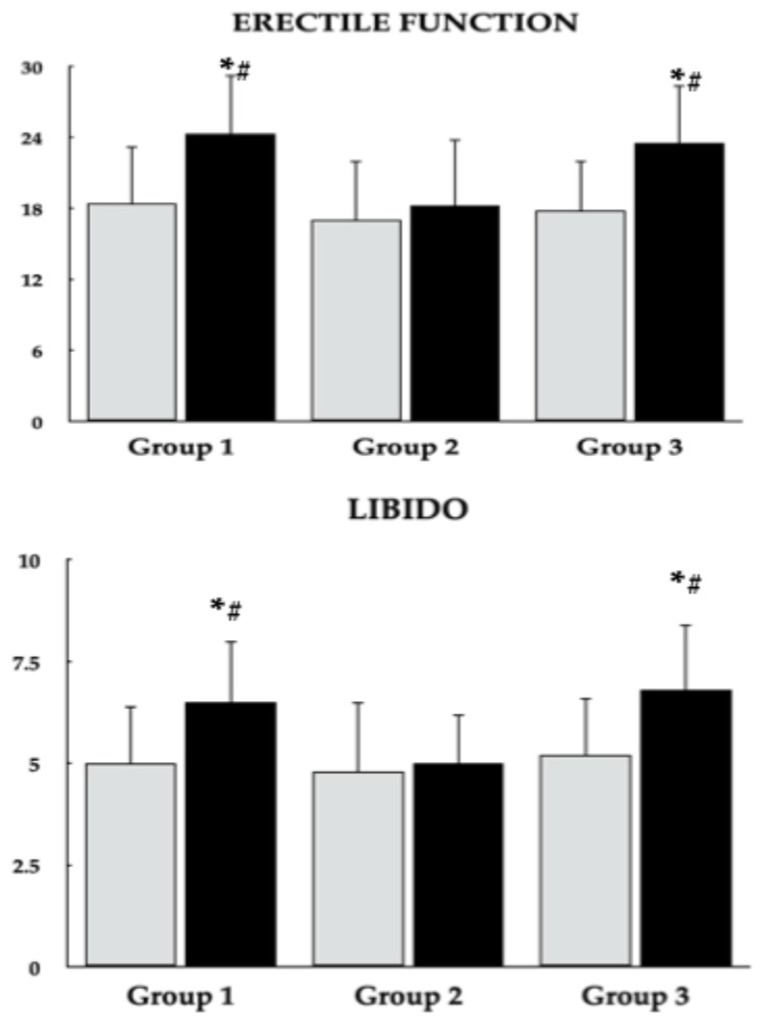
Effect of metformin on domain scores for erectile function and libido (sexual desire) in men with antipsychotic-induced hyperprolactinemia across different vitamin D statuses. Data are presented as mean ± standard deviation. Gray bars represent values at baseline; black bars represent values at study completion. Group 1: men with normal vitamin D status not receiving vitamin D preparations; Group 2: men with low vitamin D status not receiving vitamin D preparations; Group 3: men with normal vitamin D status due to vitamin D supplementation. * *p* < 0.05 vs. the corresponding value in group 2. ^#^ *p* < 0.05 vs. baseline values within the same group.

Across all study groups, metformin treatment led to reductions in fasting plasma glucose, HbA_1c_, and HOMA-IR, although these metabolic improvements were less marked in group 2 than in the other groups. In groups 1 and 3, metformin was additionally associated with decreases in both total and monomeric PRL levels, as well as increases in LH and testosterone concentrations. In contrast, concentrations of 25OHD, macroprolactin, FSH, TSH, and IGF-1 did not change over the course of the study. Significant intergroup differences were observed in post-treatment values of 25OHD, fasting glucose, HbA_1c_, HOMA-IR, total and monomeric PRL, LH, and testosterone (Figure 2, Figure 3 and Figure 4, Table 3). Moreover, metformin treatment increased domain scores for erectile function and sexual desire in groups 1 and 3, whereas no changes were observed in group 2. At the end of the study, scores for both domains were higher in groups 1 and 3 than in group 2 (Figure 5). Body mass index values on the last day of the study were 24.7 ± 5.3 kg/m^2^ in group 1, 26.5 ± 5.9 kg/m^2^ in group 2, and 24.9 ± 5.7 kg/m^2^ in group 3. These differences were not statistically significant, either between groups or relative to baseline values.

The magnitude of the metformin-induced change in plasma PRL levels showed a positive association with baseline PRL concentrations across all study groups (total PRL: group 1, r = 0.482, *p* < 0.0001; group 2, r = 0.452, *p* = 0.0002; group 3, r = 0.467, *p* = 0.0001; monomeric PRL: group 1, r = 0.525, *p* < 0.0001; group 2, r = 0.506, *p* < 0.0001; group 3, r = 0.536, *p* < 0.0001). In group 2, treatment-related changes in PRL were positively correlated with baseline 25OHD concentrations (total PRL: r = 0.398, *p* = 0.0008; monomeric PRL: r = 0.431, *p* = 0.0004). Moreover, baseline 25OHD levels in this group were positively associated with improvements in markers of glucose homeostasis, including fasting plasma glucose (r = 0.295, *p* = 0.0465), HbA_1c_ (r = 0.351, *p* = 0.0181), and HOMA-IR (r = 0.382, *p* = 0.0086). In groups 1 and 3, increases in LH were positively associated with reductions in PRL concentrations (total PRL: group 1, r = 0.325, *p* = 0.0295; group 3, r = 0.311, *p* = 0.0403; monomeric PRL: group 1, r = 0.368, *p* = 0.0125; group 3, r = 0.380, *p* = 0.0098), as well as with increases in testosterone levels (group 1, r = 0.488, *p* < 0.0001; group 3, r = 0.472, *p* = 0.0001). In groups 1 and 3, positive correlations were also observed between the effect of metformin on testosterone levels and domain scores for erectile function (group 1: r = 0.320, *p* = 0.0298; group 3: r = 0.345, *p* = 0.0204) and sexual desire (group 1: r = 0.515, *p* < 0.0001; group 3: r = 0.503, *p* < 0.0001). In neither treatment group were the hormonal effects of metformin correlated with daily vitamin D intake.

## 4. Discussion

At baseline, all study groups exhibited markedly elevated PRL levels, which, due to the matching procedure, did not differ between groups. Correlation analysis indicated that hyperprolactinemia in the study population was associated with hypofunction of the hypothalamic–pituitary–testicular axis. Given the established association between low testosterone levels and bone loss, hypolibidemia, and impaired erection [40], PRL excess may explain the increased prevalence of osteoporosis and sexual dysfunction in long-term users of antipsychotic medications [10,41]. Impaired sexual functioning was also observed in our study participants. Therefore, despite its relatively oligosymptomatic presentation in men [42], antipsychotic-induced hyperprolactinemia warrants targeted therapeutic intervention.

Although the metformin dose used in the study was high, such doses are employed in the treatment of type 2 diabetes, particularly in cases characterized by significant insulin resistance, which is typical of patients with schizophrenia receiving antipsychotic treatment [19,43]. Moreover, its safety has been demonstrated in the treatment of conditions other than diabetes, including the reduction in body weight in individuals with obesity or overweight [44], as well as in the prevention of complications related to preeclampsia [45]. The choice of this dose was also supported by findings from our previous studies, in which we observed that the effects of metformin at the level of the anterior pituitary gland, including its influence on PRL secretion, depended on the administered dose and were most pronounced at a daily dose of 3 g [3,8]. The present study confirms the safety of a daily dose of 3 g of metformin. Adverse events led to treatment discontinuation in only two patients (2.9%); these events were limited to the gastrointestinal tract and resolved completely in both patients after discontinuation of therapy. The beneficial effects observed in individuals with normal vitamin D status, together with the safety profile of the treatment, support the potential benefits of high-dose metformin therapy in men with schizophrenia and concomitant disturbances in glucose homeostasis.

High baseline PRL levels adequately explain the significant reduction in PRL concentrations observed in patients with sufficient 25OHD levels. Consistent with this interpretation, metformin-induced changes in PRL levels in men with normal vitamin D status were positively correlated with pretreatment PRL concentrations. Another noteworthy finding was that the decrease in PRL concentrations resulted from a reduction in monomeric PRL, whereas concentrations of polymeric PRL forms remained unchanged. Given that macroprolactin arises from extrapituitary polymerization of monomeric PRL [46], this pattern suggests that metformin lowers PRL primarily by suppressing the secretory activity of pituitary lactotrophs. Although direct experimental evidence in PRL-producing cells is unavailable, previous studies have demonstrated that metformin inhibits gonadotropin release stimulated by gonadotropin-releasing hormone and activin in primary pituitary cell cultures [47]. It should be underlined that macroprolactin levels in our study were normal, partially due to the exclusion of men fulfilling the criteria for macroprolactinemia. However, correlations between the degree of PRL reduction and pretreatment values were stronger for monomeric PRL than for total PRL. This observation may justify preferential measurement of monomeric rather than total PRL, at least in patients with concomitant type 2 diabetes, who have been found to be more predisposed to macroprolactin excess than individuals with normal glucose homeostasis [48].

The key observation of this study was the lack of a PRL-lowering response to metformin in men with untreated vitamin D deficiency or insufficiency. In addition, the magnitude of the treatment effect on both total and monomeric PRL in individual patients was positively correlated with baseline 25OHD concentrations. Comparable associations have previously been described in women of reproductive age [28]. These findings allow several conclusions to be drawn. First, reduced vitamin D status appears to diminish the PRL-suppressive effects of metformin regardless of sex. Second, the extent to which vitamin D deficiency compromises metformin activity in lactotropic cells depends on the severity of the deficiency. Third, these observations may help explain quantitative discrepancies among previous studies and, given the widespread prevalence of vitamin D deficiency, may also account for the generally modest average reduction in PRL observed after metformin treatment [7,8,11,13]. Finally, routine assessment of circulating 25OHD levels appears justified in all individuals receiving antipsychotics that increase PRL concentration, or at least in those with indications for metformin treatment.

The observed differences in the effects of metformin cannot be explained by differences in the baseline severity of schizophrenia, by changes in symptom severity during treatment, or by differences in antipsychotic therapy. The first of these parameters was assessed using the PANSS-6 scale, which is a psychometrically valid measure of the core positive and negative symptoms of schizophrenia [49]. The mean score on this scale was similar across all groups and did not change in response to metformin treatment. Moreover, it did not correlate with the effect of metformin on the hormones assessed in the study. The lack of significant changes during the study largely resulted from the inclusion of individuals in remission, with the majority of participants remaining in remission throughout the study period, except for two individuals who discontinued participation prematurely. Furthermore, the absence of an association between the effects of metformin and the antipsychotic treatment used is supported by the study design, which included only individuals receiving antipsychotic medications with a well-documented propensity to increase prolactin levels. These medications were administered at stable doses for at least three months prior to study enrollment and throughout the entire duration of the study. Although study participants were treated with different antipsychotic agents—reflecting current clinical practice in the treatment of schizophrenia [50]—no differences were observed between the groups in the proportion of patients receiving first-generation versus second-generation antipsychotics, nor were there differences in other aspects of antipsychotic treatment.

Another notable observation was that the investigational drug exerted comparable effects in two subgroups of patients with normal vitamin D status: those not receiving vitamin D supplements and those treated with exogenous vitamin D following prior deficiency or insufficiency. Importantly, dietary vitamin D intake showed no association with the effect on PRL levels. These findings indicate that the effect of metformin on the secretory activity of lactotropic cells is determined by current vitamin D status rather than by vitamin D supplementation per se. Accordingly, the inhibitory influence of metformin on this population of anterior pituitary cells appears to be transient and reversible with adequate restoration of vitamin D levels. This suggests that overall vitamin D sufficiency, rather than a pharmacokinetic interaction with exogenous vitamin D, underlies normalization of the PRL response to metformin. Collectively, these results highlight the need for vitamin D supplementation even in cases of mild insufficiency.

The reduction in PRL levels was accompanied by increases in LH and testosterone. Given the adverse consequences of low testosterone levels in males [40], this effect may have clinical relevance. The most plausible explanation for our findings is that stimulation of the hypothalamic–pituitary–testicular axis occurred secondary to the improvement in PRL secretion. This interpretation is supported by the observation that metformin-induced changes in PRL and LH were positively correlated, and that similar positive associations were noted between the effects of the study drug on LH and testosterone. In theory, metformin may also exert a direct effect on testicular testosterone production. The drug has previously been shown to increase circulating testosterone levels in men with type 2 diabetes [51], an effect that was likely mediated through its action on testicular steroidogenesis [52]. Moreover, vitamin D deficiency and insufficiency in men may predispose to reduced circulating testosterone levels, which have been reported to increase in response to calcitriol treatment [53]. However, a predominant testicular effect would be expected to elicit a compensatory reduction in LH secretion, which is inconsistent with our observations. Thus, even if metformin directly stimulates testosterone production in men with iatrogenic hyperprolactinemia, this effect appears to be less significant than its action at the pituitary level.

The clinical consequence of increased testosterone levels was an improvement in sexual function, observed exclusively in groups with normal vitamin D status. The association with strengthened activity of the hypothalamic–pituitary–gonadal axis was supported by positive correlations between the effect of metformin on testosterone levels and scores in both domains assessed by the IIEF-15 questionnaire [38]. Stronger correlations with libido than with erectile function underscore the greater role of testosterone in regulating the former component of sexual response, whereas erectile function largely depends on nonhormonal mechanisms, particularly vascular flow and neural regulation [54]. It should be emphasized, however, that even in groups showing improvement, sexual function at the end of the study remained substantially impaired. This reflects the detrimental effects of the underlying disease and antipsychotic medications, which disrupt sexual activity partially independent of increases in prolactin levels [55,56].

In view of the present findings and previously published data [28], vitamin D and its metabolites do not appear to mediate the effect of metformin on circulating PRL concentrations. Metformin treatment was not associated with changes in 25OHD levels in either study group. This observation is consistent with the findings of Out et al. [57], who reported stable 25OHD concentrations in patients with type 2 diabetes after 16 months of therapy with high-dose metformin (2.55 g/day). These convergent data suggest that metformin does not interfere with vitamin D synthesis or metabolic pathways. Earlier studies have also shown that long-term administration of calcitriol, the biologically active form of vitamin D, does not alter PRL secretion [58,59]. Our own unpublished data further support this notion, indicating no effect of vitamin D supplementation on circulating PRL levels. Evidence supporting the reverse interaction is likewise limited. Metformin is not metabolized and is eliminated unchanged via renal excretion, resulting in a low likelihood of clinically relevant pharmacokinetic interactions, which are largely confined to compounds sharing cation transport systems [43]. To our knowledge, no studies have demonstrated that vitamin D or its derivatives affect the absorption, distribution, metabolism, or excretion of metformin.

Thus, two biological systems appear most plausibly involved in potential cross-talk between metformin and vitamin D: the pituitary adenosine monophosphate-activated protein kinase (AMPK) signaling pathway and the tuberoinfundibular dopaminergic axis. AMPK is a key intracellular target mediating the metabolic effects of metformin across various animal species and in humans [60], and pituitary studies have demonstrated that this pathway also underlies metformin-induced alterations in gonadotropin secretion in rats [47]. Moreover, findings from non-pituitary tissues, including the kidney and prostate cancer models in rodents and humans, suggest that combined exposure to metformin and vitamin D produces cooperative biological effects that are mechanistically linked to AMPK activation [61,62]. Additional evidence suggests that metformin and vitamin D may exert additive effects on the tuberoinfundibular dopaminergic system, the principal inhibitory regulator of PRL secretion [63]. Vitamin D receptors are expressed in the rodent pituitary [64], a brain region shown to accumulate substantial amounts of metformin in rats [65]. Furthermore, calcitriol has been reported to stimulate dopamine synthesis and release in various dopaminergic neuronal populations in rats [66]. Finally, enhanced endogenous hypothalamic dopaminergic tone has been identified as a mediator of metformin action in insulin-resistant patients [67]. 

In contrast to the lack of any effect on PRL concentrations, uncorrected vitamin D deficiency or insufficiency did not abolish the beneficial effect of metformin on carbohydrate metabolism, although this effect was less pronounced than in individuals with adequate vitamin D status. These results confirm the efficacy of metformin in cases of prediabetes or type 2 diabetes in patients with schizophrenia receiving antipsychotic medications [68,69]. Furthermore, they underscore the importance of assessing vitamin D status in this patient population and correcting potential deficiencies. Measurement of 25OHD should always be recommended before adjusting hypoglycemic therapy in patients demonstrating an insufficient response to metformin. The absence of correlations between baseline PRL levels and pretreatment values of glucose, HbA_1c_, and HOMA-IR argues against a primary role for hyperprolactinemia in the development of type 2 diabetes and prediabetes in the population evaluated in our study. Furthermore, the lack of similar correlations during metformin therapy suggests that, in men, the PRL-lowering and metabolic effects of metformin are independent of one another. The lack of parallelism between the hormonal and metabolic effects of metformin under conditions of low vitamin D status may be explained by differences in tissue expression of AMPK. AMPK expression is higher in the liver, skeletal muscle, and adipose tissue—where it plays a key role in metformin-mediated regulation of glucose homeostasis—than in the pituitary [60,70]. Despite improvements in glucose homeostasis and previously reported benefits in preventing antipsychotic-induced weight gain [69,71], our study did not observe a statistically significant reduction in body mass index in metformin-treated patients, even among those with sufficient vitamin D. This finding may reflect the relatively short duration of the study, the initiation of metformin several months after starting antipsychotic therapy, and the inclusion of a minimum 12-week lifestyle modification period before enrollment.

Interpretation of the present findings is limited by several methodological considerations. Although the sample size in each group met prespecified requirements, the relatively small number of participants and the short observation period suggest that the results should be regarded as primarily hypothesis-generating and warrant validation in larger studies. The study included a male population spanning a wide age range, which limited the ability to identify differences among specific age groups. A delayed effect of non-pharmacological interventions cannot be entirely excluded. As all baseline 25OHD concentrations remained below 150 nmol/L, the potential pituitary effects of metformin in patients with higher concentrations merit further investigation. The study design inherently carries a risk of residual confounding and selection bias, which may have influenced the observed outcomes. Despite methodological safeguards applied during study design and data analysis, residual regression toward the mean may still have affected the results [72]. Macroprolactin analysis was performed using a precipitation technique, whereas gel filtration chromatography is currently considered the reference standard [73]. Finally, this study did not investigate the molecular mechanisms underlying the observed findings.

## 5. Conclusions

Summing up, metformin therapy is an effective PRL-lowering strategy in males with markedly elevated PRL levels resulting from antipsychotic treatment, but only when normal vitamin D status is preserved. In the presence of untreated vitamin D deficiency or insufficiency, metformin does not influence PRL secretion; however, restoration of adequate vitamin D levels re-establishes this effect. Sufficient vitamin D status also appears to be a prerequisite for the optimal metabolic action of metformin on glucose homeostasis in hyperprolactinemic men. These observations indicate that men treated with antipsychotics who require long-term metformin therapy are likely to benefit from systematic assessment of vitamin D status and timely correction of deficiencies. Because of the matched cohort design and other protocol limitations, the findings require confirmation in randomized clinical trials. Furthermore, the results highlight the need for mechanistic studies aimed at elucidating the biological basis of the interaction between metformin and vitamin D.

## Figures and Tables

**Table 1:** Baseline characteristics of the study groups.

Variable	Group 1	Group 2	Group 3
**Number** (*n*)	23	23	24
**Age** (years)	53 ± 12	55 ± 12	52 ± 11
**Smokers** (%)/**Number of cigarettes a day** (*n*)**/Duration of smoking** (years)	61/8 ± 5/26 ± 10	52/10 ± 6/29 ± 10	58/9 ± 5/28 ± 8
**Primary or vocational/secondary/university education** (%)	26/43/30	22/39/39	25/38/38
**Employed/Blue-collar/white-collar/pink-collar workers** (%)	87/39/39/9	83/43/35/4	83/39/39/4
**Physical activity: mild/moderate/vigorous** (%)	48/43/9	43/43/13	46/42/12
**Type 2 diabetes/prediabetes (%)**	52	48	50
**PANSS-6 score**	9.8 ± 1.9	10.1 ± 2.2	9.7 ± 1.8
**First/second generation antipsychotic** (%) ^1^	26/74	30/70	29/61
**Body mass index** (kg/m^2^)	25.9 ± 5.5	27.1 ± 5.8	26.1 ± 5.3
**Systolic blood pressure** (mmHg)	128 ± 15	132 ± 16	127 ± 18
**Diastolic blood pressure** (mmHg)	84 ± 5	86 ± 7	84 ± 6
**Total daily vitamin D intake with food** (µg) ^2^	18.8 ± 6.8 *	9.2 ± 4.6	9.5 ± 3.8

Unless otherwise stated, values are reported as mean ± standard deviation. Group 1: men with normal vitamin D status not receiving vitamin D preparations; Group 2: men with low vitamin D status not receiving vitamin D preparations; Group 3: men with normal vitamin D status due to vitamin D supplementation. ^1^ Only medications documented to induce hyperprolactinemia were allowed. ^2^ Without vitamin D in tablet/capsule form. * Statistically significant (*p* < 0.05) compared with the remaining two groups.

**Table 2:** Summary of mean and individual daily antipsychotic doses.

Drugs	Group 1		Group 2		Group 3	
	Mean Dose (mg)	Individual Doses (mg)	Mean Dose (mg)	Individual Doses (mg)	Mean Dose (mg)	Individual Doses (mg)
Perazine (*n* = 2, *n* = 2, *n* = 3)	175	150 and 200	150	100, 200	167	150, 150 and 200
Haloperidol (*n* = 2, *n* = 2, *n* = 2)	2	2 and 2	3	2 and 4	2.5	2 and 3
Flupenthixole (*n* = 2, *n* = 3, *n* = 2)	1.25	1 and 1.5	1.17	1, 1, 1.5	1.5	1 and 1
Risperidone (*n* = 13, *n* = 11, *n* = 12)	3.75	2, 2, 2, 3, 4, 4, 4, 4, 4, 4, 5, 6 and 6	3.91	1, 2, 2, 4, 4, 4, 4, 5, 5, 6 and 6	3.83	2, 2, 2, 4, 4, 4, 4, 4, 4, 4, 6 and 6
Amisulpride (*n* = 4, *n* = 5, *n* = 5)	500	400, 400, 600 and 600	440	200, 400, 400, 600 and 600	480	400, 400, 400, 600 and 600

The successive values of *n* in the first column correspond to the number of patients in groups 1, 2, and 3, respectively. Group 1: men with normal vitamin D status not receiving vitamin D preparations; Group 2: men with low vitamin D status not receiving vitamin D preparations; Group 3: men with normal vitamin D status due to vitamin D supplementation.

**Table 3:** Percentage changes from baseline in the assessed parameters.

Variable	Group 1	Group 2	Group 3
25-hydroxyvitamin D	2 ± 10	4 ± 12	1 ± 8
Fasting glucose	−23 ± 8 *	−11 ± 7	−24 ± 9 *
HbA_1c_	−15 ± 8 *	−8 ± 8	−15 ± 7 *
HOMA-IR	−42 ± 18 *	−25 ± 20	−50 ± 23 *
Total prolactin	−29 ± 14 *	3 ± 16	−28 ± 15 *
Monomeric prolactin	−31 ± 12 *	4 ± 14	−28 ± 15 *
LH	48 ± 20 *	−8 ± 23	40 ± 21 *
Testosterone	25 ± 15 *	−4 ± 16	30 ± 19 *
Macroprolactin	4 ± 25	−10 ± 24	−8 ± 24
FSH	11 ± 18	3 ± 23	10 ± 28
TSH	−17 ± 18	−12 ± 19	−17 ± 20
ACTH	36 ± 35	43 ± 38	42 ± 40
IGF-1	8 ± 40	−11 ± 42	8 ± 43

Data are presented as mean ± standard deviation. Group 1: men with normal vitamin D status not receiving vitamin D preparations; Group 2: men with low vitamin D status not receiving vitamin D preparations; Group 3: men with normal vitamin D status due to vitamin D supplementation. * *p* < 0.05 vs. the corresponding value in group 2.

## Data Availability

The data that support the findings of this study are available from the corresponding author upon reasonable request due to privacy.

## Supplementary Materials

The following supporting information can be downloaded at: https://www.mdpi.com/article/10.3390/nu18071062/s1, Table S1: Mean calorie and macronutrient intake among the study groups.
