# Supplementary material for: Vitamin D Adequacy Conditions the Prolactin-Suppressive Effect of Metformin in Men Receiving Prolactin-Elevating Medications
Source: Nutrients. 2026 Mar 26;18(7):1062. doi: 10.3390/nu18071062 (PMC13075181; doi:10.3390/nu18071062)
Supplement: Supplementary file 1 [file nutrients-18-01062-s001.zip › nutrients-4172519-supplementary.pdf]

Supplementary Table S1. Mean calorie and macronutrient intake among the study groups

Variable	Group 1	Group 2	Group 3
Mean total daily calorie intake (kcal/kg)			
Before the study	31.2 ± 2.8	30.8 ± 3.0	31.0 ± 2.8
During the study	31.4 ± 2.5	30.9 ± 2.6	31.2 ± 3.1
Mean daily carbohydrate intake (g)			
Before the study	355 ± 46	367 ± 50	346 ± 48
During the study	364 ± 40	372 ± 42	356 ± 55
Mean daily lipid intake (g)			
Before the study	79 ± 6	82 ± 7	81 ± 8
During the study	78 ± 5	81 ± 5	79 ± 7
Mean daily protein intake (g)			
Before the study	95 ± 14	98 ± 11	98 ± 16
During the study	93 ± 16	97 ± 9	100 ± 15
Mean daily fiber intake (g)			
Before the study	24 ± 4	22 ± 5	23 ± 4
During the study	25 ± 3	24 ± 5	25 ± 4

Data are presented as mean ± standard deviation. Group 1: men with normal vitamin D status not receiving vitamin D preparations; Group 2: men with low vitamin D status not receiving vitamin D preparations; Group 3: men with normal vitamin D status due to vitamin D supplementation.
